# Midwives' approach to the prevention and repair of obstetric perineal trauma in Spain

**DOI:** 10.1002/nop2.2160

**Published:** 2024-04-25

**Authors:** Estíbaliz Laderas Díaz, Julián Rodríguez‐Almagro, Rafael Picón Rodríguez, Juan Miguel Martínez Galiano, Sandra Martínez Rodríguez, Antonio Hernández‐Martínez

**Affiliations:** ^1^ Department of Obstetrics & Gynecology La Mancha Centro General Hospital Alcázar de San Juan, Ciudad Real Spain; ^2^ Department of Nursing, Physiotherapy and Occupational Therapy, Ciudad Real Faculty of Nursing University of Castilla‐La Mancha Ciudad Real Spain; ^3^ Department of General and Digestive Surgery Santa Bárbara Hospital Puertollano, Ciudad Real Spain; ^4^ Department of Nursing Jaen University Jaen Spain; ^5^ Consortium for Biomedical Research in Epidemiology and Public Health (CIBERESP) Madrid Spain

**Keywords:** episiotomy, midwife, obstetrics, perineal tear, professional practice

## Abstract

**Aim:**

Different clinical practice guidelines include recommendations on how to prevent and repair obstetric perineal trauma, as well as the use of episiotomy. To evaluate the variability in midwives' professional practices for preventing and repairing perineal trauma, as well as the professional factors that may be associated with the restrictive use of episiotomy.

**Design:**

Observational cross‐sectional study.

**Methods:**

Three hundred five midwives completed an anonymous questionnaire developed by the authors and distributed across various midwifery scientific societies. The main outcomes measured were the frequencies of adopting specific practices related to perineal injury prevention and repair, episiotomy technique and restrictive episiotomy use (<10%). Odds ratios (OR) and adjusted odds ratios (aOR) with 95% confidence intervals were estimated.

**Results:**

Intrapartum perineal massage was not normally used by 253 (83%) of midwives, and 186 (61%) applied compresses soaked in warm water to the perineum. Regarding episiotomy, there was a great deal of variability, noting that 129 (42.3%) adopted a restrictive use of this procedure, 125 (41%) performed it between 10% and 20%, while 51 midwives (16.7%) performed it in more than 20% of cases. In addition, 165 (54.1%) midwives followed an incision angle of 60º. Concerning tears, 155 (50.8%) usually sutured first‐degree tears and 273 (89.5%) always sutured second‐degree tears. Midwives attending home births (aOR = 6.5; 95% CI: 2.69–15.69), working at a teaching hospital (aOR = 3.69; 95% CI: 1.39–9.84), and the ones who recently completed their professional training (aOR = 3.58; 95% CI: 1.46–8.79) were significantly more likely to adopt a restrictive use of episiotomy.

**Conclusions:**

There is a significant variability in Spanish midwives' practices for preventing and repairing perineal tears. Moreover, the restrictive use of episiotomy is associated with midwives attending home births, working in teaching hospitals and having recent professional training.

**Patient or Public Contribution:**

No patient or public contribution.

## 1 INTRODUCTION

Genital tract trauma, resulting from episiotomy, spontaneous obstetric lacerations or both, is a common occurrence during childbirth (Abedzadeh‐Kalahroudi et al., 2019), with incidences as high as 85% in vaginal births (Frohlich & Kettle, 2015). The occurrence of second‐degree tears ranges between 31% and 62%, whereas third and fourth‐degree tears have a reported prevalence of approximately 2.9% (Thiagamoorthy et al., 2014) to 3.1% (Pergialiotis et al., 2020) in vaginal births. The associated morbidity, particularly in cases affecting the anal sphincter and rectal mucosa, is significant (Abdelhakim et al., 2020). These injuries are linked to various complications, including infection rates ranging from 0.1% to 23.6%, and suture dehiscence occurring in 0.21% to 24.6% of cases (Jones et al., 2019). Higher degree tears are correlated with increased frequency of these complications. Additionally, these injuries are associated with postpartum dyspareunia (17%–36%) (Alligood‐Percoco et al., 2016) at 6 months postpartum, stress urinary incontinence (30%) (Shinozaki et al., 2022) and faecal incontinence (5.5%) (Zizzi et al., 2017).

In response to the significant issues associated with perineal trauma post‐childbirth, various interventions have been implemented for prevention and minimization (Aasheim et al., 2017; Fretheim et al., 2013; Kopas, 2014; Marcos‐Rodríguez et al., 2023; Martínez et al., 2021). Notably, the avoidance of routine episiotomy is strongly advocated in most guidelines (World Health Organization, 2018), as restrictive episiotomy practices have not been linked to foetal acidosis or adverse infant outcomes (FIGO Safe Motherhood and Newborn Health (SMNH) Committee, 2012; Friedman et al., 2015; Hidalgo‐Lopezosa et al., 2016). Consequently, the prevalence of routine episiotomy has been decreasing, though its use and overuse persist in many hospitals and among various midwives (Escuriet et al., 2015).

In light of this evidence, numerous scientific societies and agencies have developed clinical practice guidelines (CPGs) and protocols to standardize recommendations on perineal trauma prevention and episiotomy practice. In 2018, the World Health Organization (WHO) published recommendations for women's care (World Health Organization, 2018), as did the National Institute for Health and Care Excellence (NICE) (National Institute for Health and Care Excellence, 2014b). In Spain, the Ministry of Health, Social Policy and Equality published the CPG on Normal Childbirth in 2010 (Ministerio de Sanidad y Política Social, 2010). The aim of these guidelines and protocols is to reduce variability in care practices and to promote professional adherence to techniques or recommendations that mitigate perineal damage during the second stage of labour.

Diverse strategies have been developed internationally aimed at mitigating severe perineal trauma, yielding outcomes with significant promise. These strategies encompass the initiation of interdisciplinary educational modules intended to bolster the confidence of midwifery practitioners in refraining from episiotomy procedures (Frost et al., 2016), eschewing the semi‐reclined birthing posture in favour of training in controlled expulsive efforts and facilitating the spontaneous delivery of the shoulders (Basu et al., 2016), along with the application of prenatal perineal massage, manual safeguarding and the utilization of EPISCISSORS‐60 to ascertain the precise episiotomical angle (Mohiudin et al., 2018). Particularly, the research conducted by Borrman et al. (2020) merits attention, wherein a diminution in the incidence of severe perineal trauma was observed subsequent to the application of several aforementioned interventions, with sustained efficacy observed over a 2‐year period.

Despite the established recommendations and the success of certain strategies in reducing perineal trauma, the preliminary step prior to identifying the most suitable strategies for implementation in our context involves understanding the current state of these practices within our environment. Investigating this topic could offer valuable insights for current clinical practice and underscore areas necessitating enhancement in professional training. This could considerably improve adherence to evidence‐based recommendations.

Thus, the objective of this study was to assess the care practices of Spanish midwives in preventing and repairing perineal trauma, as well as to identify professional profile factors associated with the restrictive practice of episiotomy.

## 2 METHOD

### 2.1 Design

This study was a cross‐sectional observational analysis focused on midwives practicing in Spain during 2021.

### 2.2 Sample and settings

The estimated sample size was based on a reference population of 9593 active midwives (Instituto Nacional de Estadística, INEbase, 2020), assuming a 50% prevalence of the factor investigated (adopted due to the multi‐response nature of the questionnaire and as a conservative estimate), an absolute error of 6%, a 10% replacement rate and a 95% confidence level. This calculation yielded a minimum required sample of 289 midwives.

### 2.3 Measures

The study employed a self‐developed, anonymous questionnaire comprising 27 closed questions: three on sociodemographic data, six on professional activity and work environment and 18 on techniques for perineal protection and repair during the second stage of labour.

Collected variables included:

- Sociodemographic variables: age groups (>25, 26–35, 36–45, 46–55, 56–65 years) and sex were used as independent variables.
- Employment‐related variables: year of midwifery training completion, employment in public/private centres, involvement in home births, work in primary care, annual birth rate at their hospital and whether their institution trains obstetric professionals.

As dependent variables, the study inquired about various methods of perineal protection and repair. This included questions on suturing techniques, the application of warm compresses, intrapartum perineal massage, active perineal protection, manual control of head deflection, episiotomy procedures and techniques for suturing different types of tears.

**FIGURE 1:**
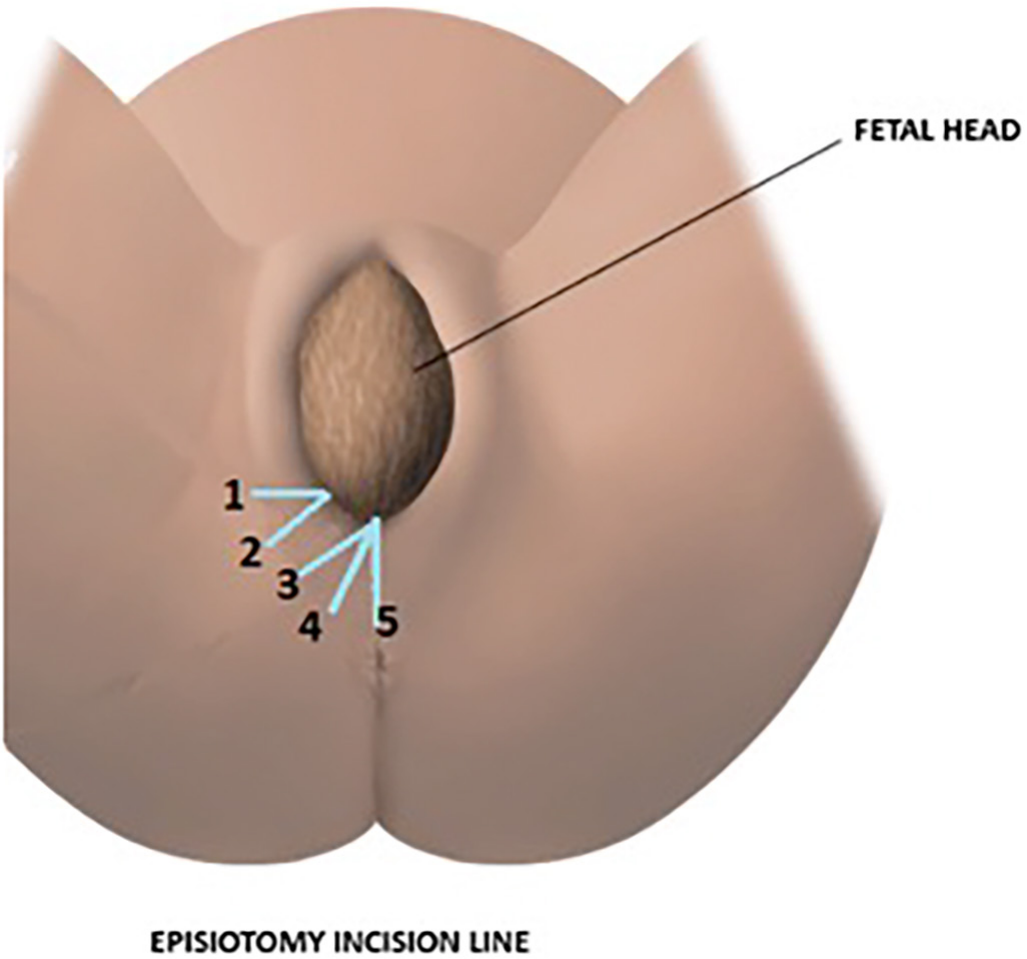
Commonly used incision angles.

The midwives were also presented with a figure showing different incision angles to be usually adopted for episiotomy and asked to choose the one used in their own practice (Figure 1).

The primary dependent variable was the practice of restrictive episiotomy (defined as a rate under 10%), following the criteria of Blanc‐Petitjean et al. (2020) and WHO targets (Technical Working Group, 1997).

### 2.4 Data collection

The questionnaire was disseminated via email through various professional and scientific midwifery organizations, including the Federation of Spanish Midwives Associations (FAME) and the National Association of Midwives of Spain, alongside regional associations.

Midwives received an information sheet about the study and instructions for completing the questionnaire. A contact email was provided for any queries.

### 2.5 Ethical considerations

This study was approved by the Clinical Research Ethics Committee (CEIC) of Hospital Mancha‐Centro with protocol number 194‐C.

### 2.6 Statistical analysis

The analysis involved calculating absolute and relative frequencies for categorical variables and mean with standard deviation for quantitative variables. This was followed by bivariate and multivariate analyses, with the restrictive episiotomy practice as the main dependent variable and professional profile characteristics as independent variables. Odds ratios (OR) and adjusted odds ratios (aOR) with 95% confidence intervals were estimated (Chen et al., 2010). All analyses were conducted using SPSS 28.0.

## 3 RESULTS

### 3.1 Professional and work environment characteristics

A total of 305 midwives participated, of whom 276 (90.5%) were women. Regarding the age of the midwives, 171 (56.1%) were between 26 and 35 years old, 296 (97%) worked at a public facility, 171 (56.8%) completed their residency training after 2015. Home births were attended by 35 (11.5%) of the midwives. Regarding their work centre, 263 (86.2%) worked in hospitals with resident training programmes, and were in hospitals handling 1000–3000 births annually. Detailed characteristics are presented in Table 1.

**TABLE 1:** Sociodemographic characteristics and professional profile.

Variable	*n* (%) (*n* = 305)
Age	
≤25 years	19 (6.2)
26–35 years	171 (56.1)
36–45 years	71 (23.3)
46–55 years	35 (11.5)
56–65 years	9 (3.0)
Gender	
Male	29 (9.5)
Female	276 (90.5)
Working at a public centre	
No	9 (3.0)
Yes	296 (97.0)
Working at a private centre	
No	270 (88.5)
Yes	35 (11.5)
Working in primary care	
No	193 (63.3)
Yes	42 (13.8)
Sometimes	70 (23.0)
Teaching hospital	
No	42 (13.8)
Midwives only	16 (5.2)
Gynaecologists only	7 (2.3)
Both specialties	240 (78.7)
Hospital size	
Up to 1000 births per year	69 (22.6)
1000–3000 births per year	180 (59.0)
More than 3000 births per year	56 (18.4)
Time since completion of midwifery training	
Prior to 2005	36 (12.0)
2005–2015	94 (31.2)
2015–2021	171 (56.8)
Missing data	4
Attending home births	
No	270 (88.5)
Yes	35 (11.5)

### 3.2 Practices related to the prevention of perineal trauma

**FIGURE 2:**
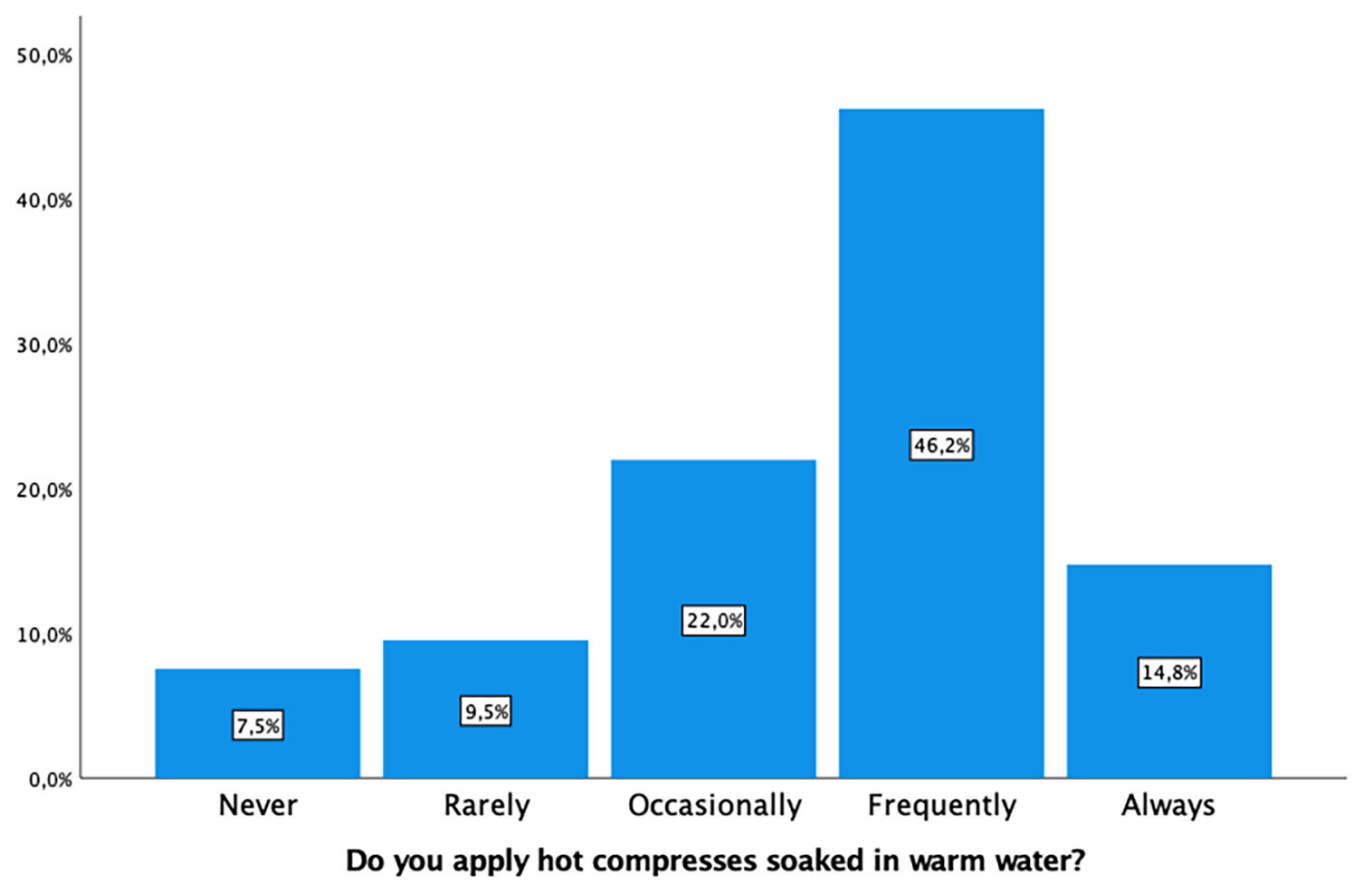
Application of hot compresses.

**FIGURE 3:**
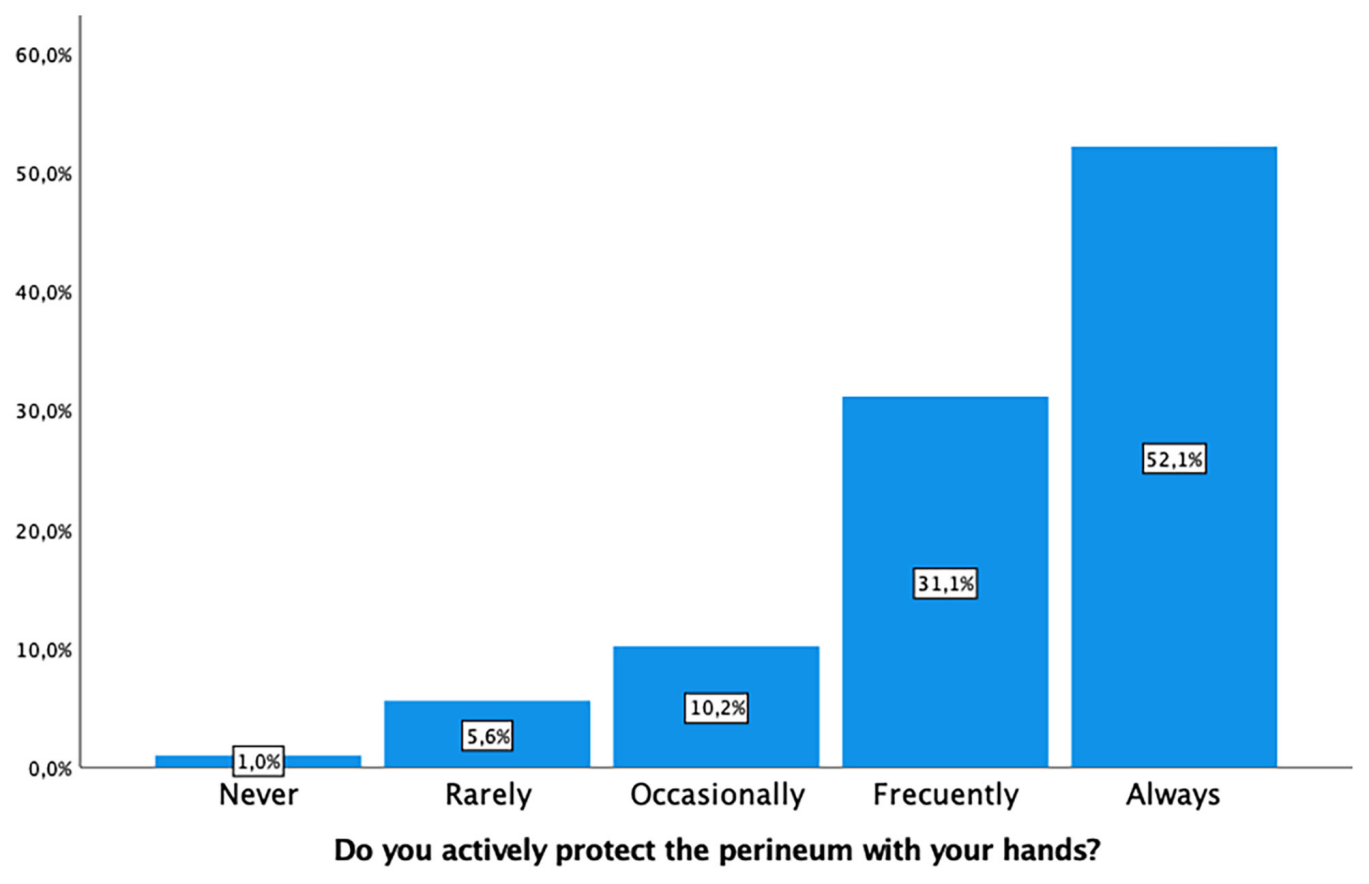
Protection of the perineum with hands.

**FIGURE 4:**
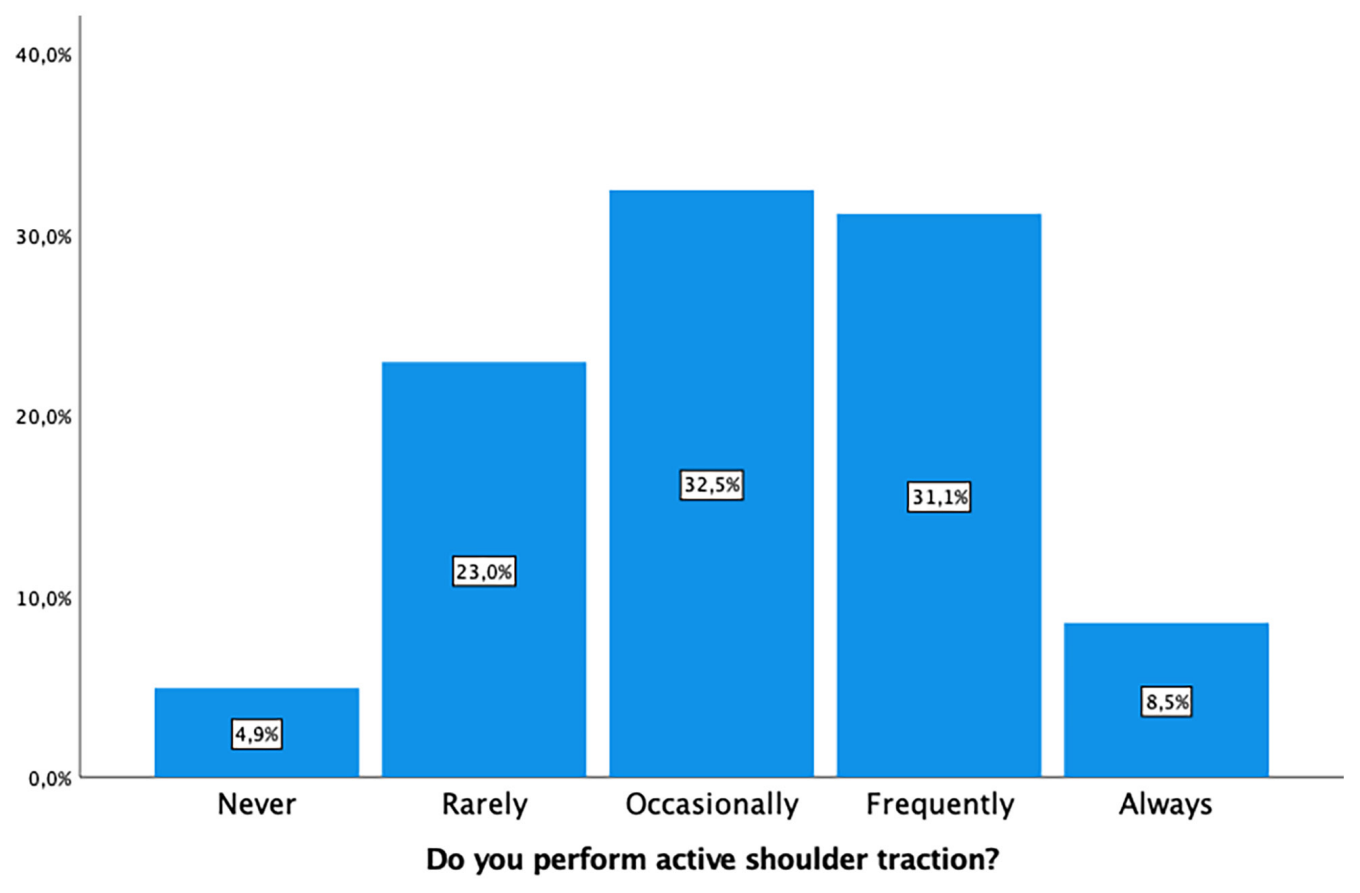
Active shoulders traction.

Intrapartum perineal massage was not applied by 253 (83%) of midwives. Warm water compresses were used by 186 (61%), and 155 (50.9%) utilized lubricants to reduce the risk of tearing (Figure 2). Active hand protection of the perineum was a common practice by 254 (83.2%) (Figure 3), as was controlling the speed of head deflection by 273 (89.5%). Encouraging the woman not to push during head deflection was advised by 260 (85.3%) midwives. Regarding active shoulder traction, 169 (55.5%) did so rarely or occasionally, while 95 (31.1%) frequently employed this technique (Figure 4). The variability in these practices is detailed in Table 2.

**TABLE 2:** Perineal protection.

Perineal protection practices	*n* (%) (*n* = 305)
Do you perform intrapartum perineal massage?	
Never	146 (47.9)
Rarely	107 (35.1)
Occasionally	39 (12.8)
Frequently	13 (4.3)
Do you apply dry hot compresses?	
Never	224 (73.4)
Rarely	53 (17.4)
Occasionally	21 (6.9)
Frequently	5 (1.6)
Always	2 (0.7)
Do you apply hot compresses soaked in warm water?	
Never	23 (7.5)
Rarely	29 (9.5)
Occasionally	67 (22.0)
Frequently	141 (46.2)
Always	45 (14.8)
Do you use lubricant in the birth canal to reduce the risk of tearing?	
Never	51 (16.7)
Rarely	45 (14.8)
Occasionally	54 (17.7)
Frequently	85 (27.9)
Always	70 (23.0)
Do you actively protect the perineum with your hands?	
Never	3 (1.0)
Rarely	17 (5.6)
Occasionally	31 (10.2)
Frequently	95 (31.1)
Always	159 (52.1)
Do you control head deflection with your hands?	
Never	2 (0.7)
Rarely	9 (3.0)
Occasionally	21 (6.9)
Frequently	90 (29.5)
Always	183 (60.0)
Do you ask the woman not to push during head deflection?	
Never	6 (2.0)
Rarely	10 (3.3)
Occasionally	29 (9.5)
Frequently	125 (41.0)
Always	135 (44.3)
Do you perform active shoulder traction?	
Never	51 (16.7)
Rarely	45 (14.8)
Occasionally	54 (17.7)
Frequently	85 (27.9)
Always	70 (23.0)

### 3.3 Episiotomy use

**FIGURE 5:**
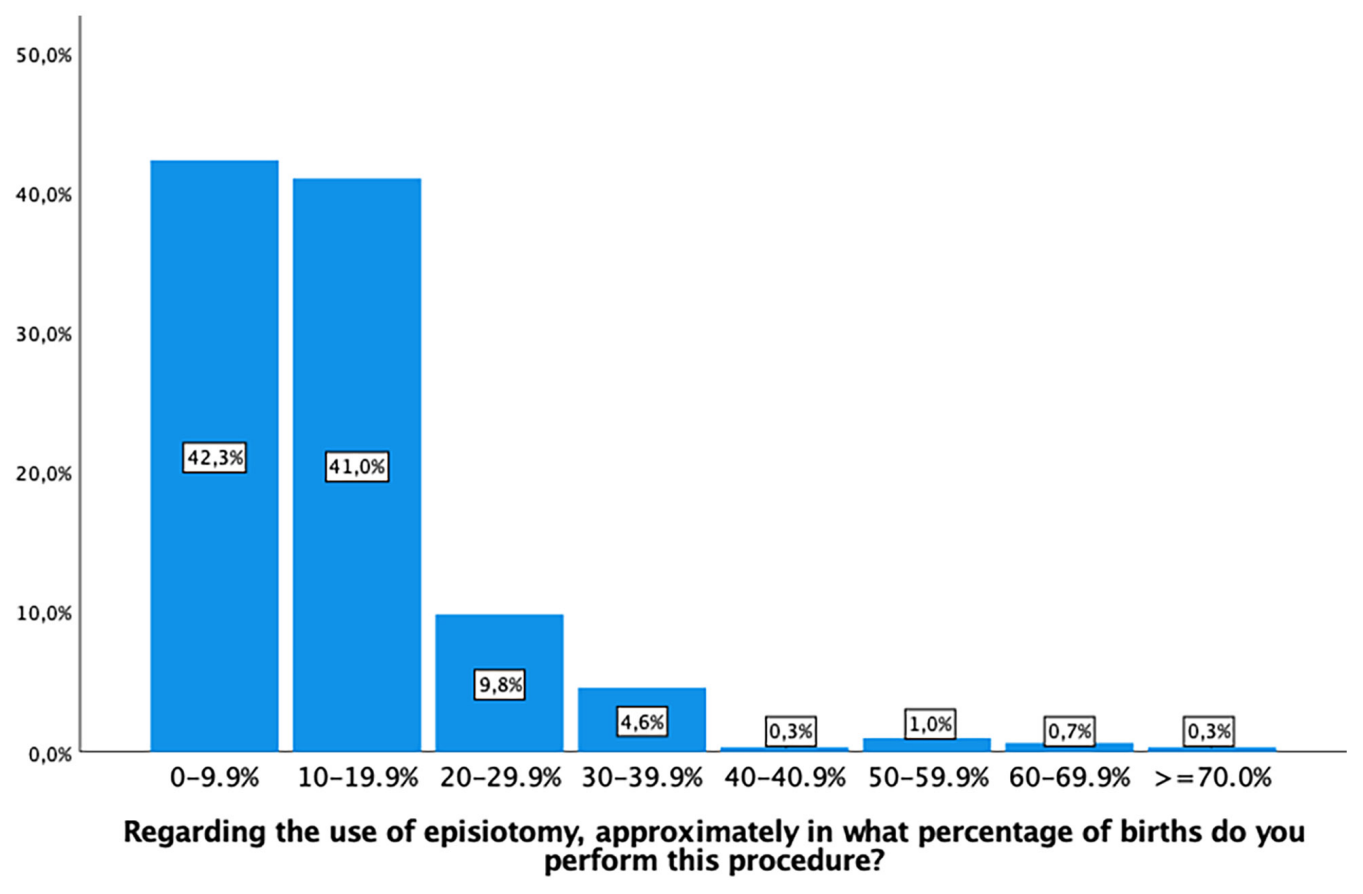
Use of episotomy.

A significant portion of the midwives (254, 83.3%) reported performing episiotomies in less than 10% of eutocic births (Figure 5). In cases of previous episiotomy, 214 (70.2%) would perform a new episiotomy over the prior incision. For previous third‐ or fourth‐degree tears, 251 (82.3%) rarely or never performed routine episiotomy. The variability observed in each of these practices can be found in Table 3. Concerning the angle of the episiotomy incision, 165 (54.1%) of participants made an incision with angle number 3, according to Figure 1. The remaining percentages were distributed in relation to angles 1, 2 and 4 (Figure 1).

**TABLE 3:** Use of episiotomy and perineal repair.

Episiotomy and perineal repair practices	*n* (%) (*n* = 305)
Regarding the use of episiotomy, approximately in what percentage of births do you perform this procedure?	
0%–9.9%	129 (42.3)
10%–19.9%	125 (41.0)
20%–29.9%	30 (9.8)
30%–39.9%	14 (4.6)
40%–49.9%	1 (0–3)
50%–59.9%	3 (1.0)
60%–69.9%	2 (0.7)
>70%	1 (0.3)
At what angle would you perform an episiotomy (Figure 1)?	
Angle n° 1	43 (14.1)
Angle n° 2	66 (21.6)
Angle n° 3	165 (54.1)
Angle n° 4	31 (10.2)
Angle n° 5	0 (0.0)
In women with previous episiotomy, if you need to perform an episiotomy again	
I never perform episiotomy	66 (21.6)
I would do it over the previous episiotomy	214 (70.2)
I would avoid the episiotomy scar and look for another location.	25 (8.2)
In women with previous third‐ and fourth‐degree tears, do you use routine episiotomy?	
Never	183 (60.0)
Rarely	68 (22.3)
Occasionally	42 (13.8)
Frequently	11 (3.6)
Always	1 (0.3)
Do you suture first‐degree tears?	
Never	2 (0.7)
Rarely	52 (17.0)
Occasionally	96 (31.5)
Frequently	121 (39.7)
Always	34 (11.1)
Do you suture second‐degree tears?	
Never	0 (0.0)
Rarely	1 (0.3)
Occasionally	3 (1.0)
Frequently	28 (9.2)
Always	273 (89.5)
Regarding repair of the vaginal mucosa, what type of technique do you use when suturing is required?	
Continuous suture	300 (98.4)
Discontinuous suture	5 (1.6)
Regarding perineal muscle repair, what type of technique do you use?	
Continuous suture	267 (87.8)
Discontinuous suture	37 (12.2)
Regarding skin repair, what type of technique do you use?	
If the muscular part is well approximated, I do not suture the skin.	15 (4.9)
Intradermal suture	209 (68.5)
Continuous intradermal suture	36 (11.8)
Discontinuous suture	42 (13.8)
Mattress suture	2 (0.7)
Others	1 (0.3)
After suturing is completed, do you perform a rectal exam?	
Never	3 (1.0)
Rarely	27 (8.9)
Occasionally	49 (16.1)
Frequently	88 (28.9)
Always	138 (45.2)

### 3.4 Perineal repair

First‐degree tears were regularly sutured by 155 (50.8%) of midwives, and 273 (89.5%) always sutured second‐degree tears. For vaginal mucosa suturing, 300 (98.4%) used a continuous technique, as did 267 (87.8%) for perineal muscle repair, and 209 (68.5%) used intradermal suturing for skin. Post‐suturing rectal examinations were frequently conducted by 226 (74.1%) midwives.

### 3.5 Variables associated with the use of restrictive episiotomy (<10%)

The multivariate analysis revealed several professional characteristics associated with the use of restrictive episiotomy. Professionals attending home births were more likely to employ this practice (aOR 6.50; 95% CI: 2.69–15.69) compared to those who did not. Similarly, professionals in hospitals with resident training (aOR 3.69; 95% CI: 1.39–9.84) and those trained post‐2015 (aOR 3.58; 95% CI: 1.46–8.79) also showed a higher tendency towards restrictive episiotomy. No significant relationship was observed between the professional's gender or the number of births attended and the use of restrictive episiotomy (Table 4).

**TABLE 4:** Restrictive episiotomy and its relationship with professional and work environment characteristics.

Variable	Practices restrictive episiotomy (<10%)		OR 95% CI	*aOR 95% CI
	No (*N* = 176), *n* (%)	Yes (*N* = 129), *n* (%)		
Gender				
Male	21 (72.4)	8 (27.6)	1 (ref.)	1 (ref.)
Female	155 (56.2)	121 (43.8)	2.05 (0.88–4.79)	2.01 (0.82–4.94)
Home birth assistance				
No	166 (61.5)	104 (38.5)	1 (ref.)	1 (ref.)
Yes	10 (28.6)	25 (71.4)	**3.99 (1.84–8.65)**	**6.50 (2.69–15.69)**
Teaching hospital				
No	34 (81.0)	8 (19.0)	1 (ref.)	1 (ref.)
Yes	142 (54.0)	121 (46.0)	**3.62 (1.62–8.12)**	**3.69 (1.39–9.84)**
Completion of training				
Prior to 2005	26 (72.2)	10 (27.8)	1 (ref.)	1 (ref.)
Between 2005 and 2015	59 (62.8)	35 (37.2)	1.54 (0.67–3.58)	1.99 (0.78–5.07)
After 2015	89 (52.0)	82 (48.0)	**2.40 (1.09–5.27)**	**3.58 (1.46–8.79)**
Number of births attended at your hospital annually				
<1000 births	47 (68.1)	22 (31.9)	1 (ref.)	1 (ref.)
1000–3000 births	98 (54.4)	82 (45.6)	1.79 (1–3.21)	1.25 (0.61–2.55)
>3000 births	31 (55.4)	25 (44.6)	1.72 (0.83–3.58)	1.21 (0.51–2.83)

*Note*: Method for interpreting the size of the OR by relating it to differences in a normal standard deviate. OR = 1.68, 3.47 and 6.71 are equivalent to Cohen's *d* = 0.2 (small), 0.5 (medium) and 0.8 (large) (Chen et al., 2010). * is multivariate analysis. Statistically significant values are bolded.

Abbreviations: aOR, Adjusted odds ratio; OR, odds ratio.

## 4 DISCUSSION

### 4.1 Prevention of perineal trauma

Within the purview of midwifery practice, essential interventions aimed at mitigating perineal trauma during the second stage of labour, that have been integrated into certain institutional protocols, encompass the application of compresses saturated with warm water, the active safeguarding of the perineum, and the regulation of the velocity at which the foetal head undergoes deflection (Borrman et al., 2020). Among these practices, the use of warm compresses stands out as particularly widespread, with more than half of the midwives employing them regularly, following recommendations from institutions like the World Health Organization (WHO) (World Health Organization, 2018), the NICE (National Institute for Health and Care Excellence, 2014b), as well as the Spanish Ministry of Health (Ministerio de Sanidad y Política Social, 2010).

When asked about the position of the hands during the second stage of birth, most of the midwives in our study chose to actively protect the perineum with one hand and control head deflection with the other one. Several studies have compared the efficacy of actively protecting the perineum with one hand while controlling the rate of head deflection with the other one, versus adopting expectant management in which the hands do not touch the perineum or the head. A Cochrane review found that expectant management reduces the rate of episiotomies but does not affect the rates of intact perineum or perineal tears (Aasheim et al., 2017). In 2015, a Systematic review of the literature concluded that both techniques are acceptable in childbirth care (Bulchandani et al., 2015). A meta‐analysis on Ritgen manoeuvre (control of foetal head deflection) was published in 2019, which concluded that it is a technique that does not protect against perineal injury and may be associated with increased postpartum pain (Aquino et al., 2020). The CPG published by NICE states that both techniques are valid and can be used to facilitate spontaneous birth (National Institute for Health and Care Excellence, 2014b).

In a similar context, to facilitate the gradual detachment of the foetal head, numerous midwives reported advising pregnant women to refrain from pushing during deflection. However, certain practices, such as intrapartum perineal massage, are discouraged by various clinical practice guidelines (Acien‐Alvarez et al., 2018; National Institute for Health and Care Excellence, 2014a). Despite this, they continue to be utilized, as evidenced by our study. As for the application of lubricants and the facilitation of active shoulder birth, there was notable variability among midwives. This variation could be attributed to the lack of conclusive evidence regarding their effectiveness in tear prevention.

### 4.2 Episiotomy

In our study, the majority of midwives reported episiotomy rates between 0% and 19.9% for eutocic births. However, a notable percentage reported rates between 20% and 39.9%. This aligns with a 2014 Spanish study, which found an average episiotomy rate of 34.9% among midwives (Hernández‐Martínez et al., 2014). These figures are lower than those estimated in developing countries, where they can exceed 40% (Woldegeorgis et al., 2022). However, they surpass the rates reported in Western countries, which are documented at 19% (Goueslard et al., 2018), 10% (Leclercq et al., 2024), and even as low as 2% (Dillon et al., 2021). Despite these variations, a decreasing trend is observed, particularly in environments where midwives exercise greater clinical autonomy. This trend is exemplified by the outcomes in a centre exclusively managed by midwives, which has reported a significant reduction in episiotomy rates to 7.4%, compared to those in traditional obstetric units (Palau‐Costafreda et al., 2023).

A Cochrane review involving 12 studies with 6177 women compared restrictive versus systematic episiotomy, finding that restrictive episiotomy is associated with lower rates of severe perineal trauma and no correlation with low Apgar scores (Jiang et al., 2017). Correspondingly, guidelines from societies like NICE recommend restrictive episiotomy practices (National Institute for Health and Care Excellence, 2014b). The WHO's 1996 objective suggested an episiotomy rate of close to 10% as reasonable (Technical Working Group, 1997), while other sources, including Hernández‐Martínez et al. (2014), suggest that rates of around 25% might be safe, consistent with recommendations by the French National College of Gynaecologists and Obstetricians (Collègue National des Gynécologues et Obtétriciens, 2005).

Rusav'y et al. (2005), in their report for the Collège National des Gynécologues et Obstétriciens Français, identify the preservation of a woman's pelvic floor as a primary rationale for performing an episiotomy. This concern underscores the significance of the procedure in safeguarding maternal health. In our study, midwives were asked whether they would routinely perform episiotomy on pregnant women who had had severe tears (III or IV) in previous births in order to protect their pelvic floor, and most of them answered no. The available evidence currently considers that this practice does not prevent the occurrence of grade III or IV tears (Hernández‐Martínez et al., 2014) and some CPGs such as the NICE guidelines do not recommend its use for this purpose (National Institute for Health and Care Excellence, 2014b).

Furthermore, regarding the angle of incision, studies have demonstrated that midline episiotomies carry a higher risk of anal sphincter injury compared to mediolateral episiotomy (Pergialiotis et al., 2014, 2020; Verghese et al., 2016). Consequently, the use of midline episiotomy is discouraged (Okeahialam et al., 2023). Our study found that no midwives reported using midline episiotomy, aligning with the current recommendations. However, there was considerable variation observed in the implementation of different mediolateral angles. The optimal incision angle is debated, with some studies advocating for a 60° angle (Kalis et al., 2011), while NICE guidelines suggest an angle between 45° and 60° (National Institute for Health and Care Excellence, 2014b). The results obtained from our study show that only half of the midwives perform mediolateral episiotomy at an angle of 60°.

### 4.3 Perineal repair

In our study, we explored midwives' practices regarding suturing these tears. While there was no consensus on suturing first‐degree tears, the majority regularly sutured second‐degree tears. Our literature review included a Randomized Controlled Trial (RCT) by (Fleming et al., 2003) which compared the outcomes of suturing versus not suturing first‐ and second‐degree perineal tears. The study found that not suturing was associated with poorer healing and inferior tissue approximation.

Furthermore, the optimal method for repairing perineal injuries has been a subject of study. A 2019 clinical trial involving 134 women across five hospitals investigated the relationship between postpartum morbidity and the suturing technique used for perineal injuries. The results indicated that continuous suturing was associated with less pain 3 months postpartum and lower rates of urinary incontinence 15 days postpartum (Martínez‐Galiano et al., 2019). Additionally, a 2020 study comparing postpartum sexual function in women who received continuous versus interrupted sutures reported that those with continuous sutures experienced earlier and more satisfactory resumption of sexual activity (Martínez‐Galiano et al., 2020). Consistent with these findings, most midwives in our study expressed a preference for continuous suturing over interrupted suturing techniques.

### 4.4 Factors influencing the adoption of restrictive episiotomy practices among midwives

This study further investigates the relationship between the professional characteristics of midwives and their engagement in restrictive episiotomy practices. A significant finding is that midwives who attend home births, known for their training and familiarity with minimal intervention during childbirth, exhibit a particularly restrictive approach to episiotomy in these typically low‐risk settings, as supported by the literature (Grünebaum et al., 2023). Another determinant of restrictive episiotomy practices is employment within a teaching hospital environment, where there exists an enhanced commitment to adhering to established guidelines. This commitment is due to the necessity for professionals to model best practices for their students, a correlation supported by additional research (Kozhimannil et al., 2017). Furthermore, the duration since obtaining midwifery qualification emerged as a related factor. Given that teaching hospitals demonstrate a higher fidelity to restrictive episiotomy guidelines, it is plausible that midwives with more recent qualifications are inclined to maintain these practices over time.

### 4.5 Strengths and limitations

One notable limitation of this study is the potential for subject selection bias. Our findings indicate that the majority of respondents were relatively young (56.1% aged between 26 and 35 years, and 56.8% obtained their midwifery degrees between 2015 and 2021). This demographic skew could be attributed to their greater familiarity or engagement with the media channels used for distributing the questionnaire. While this might limit the generalizability of our results, it also offers valuable insights into emerging trends in childbirth care practices among the newer generation of midwives, which could be indicative of future directions in this field.

### 4.6 Implications for clinical practice and research

Numerous clinical practice guidelines currently incorporate the latest evidence on optimal care strategies to reduce perineal trauma and mitigate its adverse effects. However, this study reveals that, while many midwives adhere to these recommendations, a significant number still employ practices that are not recommended, potentially due to gaps in training.

Therefore, it is crucial for health institutions and scientific societies to actively engage in the periodic updating of knowledge and skills of all professionals involved in childbirth care. This includes emphasizing the importance of applying care based on the best available evidence. By doing so, these institutions can bridge the gap between recommended and actual practices, ensuring more consistent and effective care in the prevention and management of perineal trauma.

## 5 CONCLUSIONS

This study highlights considerable variability in practices among Spanish midwives concerning the prevention and repair of perineal trauma. Notably, the practice of restrictive episiotomy (less than 10%) is predominantly observed among midwives who have undergone more recent training, those employed in teaching hospitals, and those attending home births. These findings underscore the need for enhanced training and increased awareness among midwives about the importance of implementing the best care practices based on current evidence. Addressing these training and awareness gaps is crucial for improving the standard of perineal trauma care and ensuring adherence to the most up‐to‐date clinical guidelines.

## AUTHOR CONTRIBUTIONS

Julián Rodríguez Almagro: Conceptualization, formal analysis, project administration, supervision, validation, visualization and writing—review & editing. Antonio Hernández Martínez: Conceptualization, data curation, investigation, software, supervision, validation, visualization and writing—review & editing. Rafael Picón Rodríguez: Conceptualization, methodology, validation, visualization. Sandra Martinez Rodriguez: Conceptualization, data curation, investigation, software. Estíbaliz Laderas Díaz: Conceptualization, formal analysis, methodology, resources, visualization, writing—original draft and writing—review & editing. All authors have seen and approved the manuscript. The authors abide by the copyright terms and conditions of Elsevier and the Australian College of Midwives.

## FUNDING INFORMATION

We have not required any funding to conduct this research.

## CONFLICT OF INTEREST STATEMENT

The authors declare no conflicts of interest.

## RESEARCH ETHICS COMMITTEE APPROVAL

This study was approved by the Clinical Research Ethics Committee (CEIC) of Hospital Mancha‐Centro with protocol number 194‐C on May 26th, 2021.

## Data Availability

The data that support the findings of this study are available from the corresponding author upon reasonable request.

## References

[nop22160-bib-0001] Aasheim, V. , Nilsen, A. B. V. , Reinar, L. M. , & Lukasse, M. (2017). Perineal techniques during the second stage of labour for reducing perineal trauma. The Cochrane Database of Systematic Reviews, 6(6), CD006672. 10.1002/14651858.CD006672.PUB3 28608597 PMC6481402

[nop22160-bib-0002] Abdelhakim, A. M. , Eldesouky, E. , Elmagd, I. A. , Mohammed, A. , Farag, E. A. , Mohammed, A. E. , Hamam, K. M. , Hussein, A. S. , Ali, A. S. , Keshta, N. H. A. , Hamza, M. , Samy, A. , & Abdel‐Latif, A. A. (2020). Antenatal perineal massage benefits in reducing perineal trauma and postpartum morbidities: A systematic review and meta‐analysis of randomized controlled trials. International Urogynecology Journal, 31(9), 1735–1745. 10.1007/S00192-020-04302-8 32399905

[nop22160-bib-0003] Abedzadeh‐Kalahroudi, M. , Talebian, A. , Sadat, Z. , & Mesdaghinia, E. (2019). Perineal trauma: Incidence and its risk factors. Journal of Obstetrics and Gynaecology, 39(2), 206–211. 10.1080/01443615.2018.1476473 30187786

[nop22160-bib-0004] Acien‐Alvarez, P. , Serra‐Serra, V. , Gonzalez‐Gonzalez, A. , Martinez‐Astorquiza, T. , Florido‐Navio, J. , Misguel‐Sesmero, J. R. , Ucieda‐Somoza, R. , Gonzalez‐Gonzalez, N. , Suy‐Franch, A. , Melchor‐Marcos, J. C. , Fabre‐Gonzalez, E. , Puertas‐Prieto, A. , Cararach‐Ramoneda, V. , Gallo‐Vallejo, M. , & Bajo‐Arenas, J. (2018). Documento de Consenso SEGO. Asistencia al parto . Retrieved from https://drive.google.com/file/d/0ByuDDZFNh88AZGNjMGY5NDctMDYzNi00OGM5LWJkZjMtZmM3MDc2ZmM2ZWMx/view?resourcekey=0‐TIwBq8e1Yel_wTXAXXV5QQ

[nop22160-bib-0005] Alligood‐Percoco, N. R. , Kjerulff, K. H. , & Repke, J. T. (2016). Risk factors for dyspareunia after first childbirth. Obstetrics and Gynaecology, 128(3), 512. 10.1097/AOG.0000000000001590 PMC4993626 27500349

[nop22160-bib-0006] Aquino, C. I. , Saccone, G. , Troisi, J. , Guida, M. , Zullo, F. , & Berghella, V. (2020). Is Ritgen's manoeuvre associated with decreased perineal lacerations and pain at delivery? The Journal of Maternal‐Fetal & Neonatal Medicine, 33(18), 3185–3192. 10.1080/14767058.2019.1568984 30696316

[nop22160-bib-0007] Basu, M. , Smith, D. , & Edwards, R. (2016). Can the incidence of obstetric anal sphincter injury be reduced? the STOMP experience. European Journal of Obstetrics and Gynaecology and Reproductive Biology, 202, 55–59. 10.1016/j.ejogrb.2016.04.033 27164486

[nop22160-bib-0050] Blanc‐Petitjean, P. , Meunier, G. , Sibiude, J. , & Mandelbrot, L. (2020). Evaluation of a policy of restrictive episiotomy on the incidence of perineal tears among women with spontaneous vaginal delivery: A ten‐year retrospective study. Journal of Gynecology Obstetrics and Human Reproduction, 49(8), 101870. 10.1016/j.jogoh.2020.101870 32673815

[nop22160-bib-0008] Borrman, M. J. , Davis, D. , Porteous, A. , & Lim, B. (2020). The effects of a severe perineal trauma prevention program in an Australian tertiary hospital: An observational study. Women and Birth, 33(4), e371–e376. 10.1016/j.wombi.2019.07.301 31537498

[nop22160-bib-0009] Bulchandani, S. , Watts, E. , Sucharitha, A. , Yates, D. , & Ismail, K. M. (2015). Manual perineal support at the time of childbirth: A systematic review and meta‐analysis. BJOG: An International Journal of Obstetrics & Gynaecology, 122(9), 1157–1165. 10.1111/1471-0528.13431 25976557

[nop22160-bib-0010] Chen, H. , Cohen, P. , & Chen, S. (2010). How big is a big odds ratio? Interpreting the magnitudes of odds ratios in epidemiological studies. Communications in Statistics: Simulation and Computation, 39(4), 860–864. 10.1080/03610911003650383

[nop22160-bib-0011] Collègue National des Gynécologues et Obtétriciens . (2005). L' épisitomie. Recommadations pour la practique clinique .

[nop22160-bib-0012] Dillon, S. J. , Nelson, D. B. , Spong, C. Y. , McIntire, D. D. , & Leveno, K. J. (2021). Episiotomy: Evolution of a common obstetric practice at a public hospital. American Journal of Perinatology, 41(1), 39–43. 10.1055/s-0041-1739410 34856609

[nop22160-bib-0013] Escuriet, R. , Pueyo, M. J. , Perez‐Botella, M. , Espada, X. , Salgado, I. , Gómez, A. , Biescas, H. , Espiga, I. , White, J. , Fernandez, R. , Fusté, J. , & Ortún, V. (2015). Cross‐sectional study comparing public and private hospitals in Catalonia: Is the practice of routine episiotomy changing? BMC Health Services Research, 15(1), 95. 10.1186/S12913-015-0753-Z 25889079 PMC4365515

[nop22160-bib-0014] FIGO Safe Motherhood and Newborn Health (SMNH) Committee . (2012). Management of the second stage of labor. International Journal of Gynaecology and Obstetrics, 119(2), 111–116. 10.1016/j.ijgo.2012.08.002 22980427

[nop22160-bib-0015] Fleming, V. E. M. , Hagen, S. , & Niven, C. (2003). Does perineal suturing make a difference? The SUNS trial. BJOG: An International Journal of Obstetrics & Gynaecology, 110(7), 684–689. 10.1046/J.1471-0528.2003.02353.X 12842060

[nop22160-bib-0016] Fretheim, A. , Odgaard‐Jensen, J. , Røttingen, J. A. , Reinar, L. M. , Vangen, S. , & Tanbo, T. (2013). The impact of an intervention programme employing a hands‐on technique to reduce the incidence of anal sphincter tears: Interrupted time‐series reanalysis. BMJ Open, 3(10), e003355. 10.1136/BMJOPEN-2013-003355 PMC3808759 24154515

[nop22160-bib-0017] Friedman, A. M. , Ananth, C. V. , Prendergast, E. , D'Alton, M. E. , & Wright, J. D. (2015). Variation in and factors associated with use of episiotomy. JAMA, 313(2), 197–199. 10.1001/JAMA.2014.14774 25585333

[nop22160-bib-0018] Frohlich, J. , & Kettle, C. (2015). Perineal care. BMJ Clinical Evidence, 3, 23. 10.5005/jp/books/11896_32 PMC4356152 25752310

[nop22160-bib-0019] Frost, J. , Gundry, R. , Young, H. , & Naguib, A. (2016). Multidisciplinary training in perineal care during labor and delivery for the reduction of anal sphincter injuries. International Journal of Gynaecology and Obstetrics, 134(2), 177–180. 10.1016/j.ijgo.2015.12.011 27209336

[nop22160-bib-0020] Goueslard, K. , Cottenet, J. , Roussot, A. , Clesse, C. , Sagot, P. , & Quantin, C. (2018). How did episiotomy rates change from 2007 to 2014? Population‐based study in France. BMC Pregnancy and Childbirth, 18(1), 208. 10.1186/S12884-018-1747-8 29866103 PMC5987447

[nop22160-bib-0021] Grünebaum, A. , Bornstein, E. , McLeod‐Sordjan, R. , Lewis, T. , Wasden, S. , Combs, A. , Katz, A. , Klein, R. , Warman, A. , Black, A. , & Chervenak, F. A. (2023). The impact of birth settings on pregnancy outcomes in the United States. American Journal of Obstetrics and Gynecology, 228(5), S965–S976. 10.1016/j.ajog.2022.08.011 37164501

[nop22160-bib-0022] Hernández‐Martínez, A. , Pascual‐Pedreño, A. I. , Baño Garnés, A. B. , Melero‐Jiménez, M. R. , & Molina Alarcón, M. (2014). Variabilidad en la tasa de episiotomías y su relación con desgarros perineales graves y morbilidad neonatal. Enfermería Clínica, 24(5), 269–275. 10.1016/J.ENFCLI.2014.03.005 24786985

[nop22160-bib-0023] Hidalgo‐Lopezosa, P. , Hidalgo‐Maestre, M. , & Rodríguez‐Borrego, M. A. (2016). Factores perinatales asociados con los valores de pH de sangre de cordón umbilical. Enfermería Global, 15(43), 40–50.

[nop22160-bib-0024] Instituto Nacional de Estadística, INEbase . (2020). Profesionales sanitarios colegiados . Retrieved from https://www.ine.es/jaxi/Datos.htm?tpx=49002

[nop22160-bib-0025] Jiang, H. , Qian, X. , Carroli, G. , & Garner, P. (2017). Selective versus routine use of episiotomy for vaginal birth. The Cochrane Database of Systematic Reviews, 2017(2), CD000081. 10.1002/14651858.CD000081.PUB3 PMC5449575 28176333

[nop22160-bib-0026] Jones, K. , Webb, S. , Manresa, M. , Hodgetts‐Morton, V. , & Morris, R. K. (2019). The incidence of wound infection and dehiscence following childbirth‐related perineal trauma: A systematic review of the evidence. European Journal of Obstetrics, Gynaecology, and Reproductive Biology, 240, 1–8. 10.1016/J.EJOGRB.2019.05.038 31202973

[nop22160-bib-0027] Kalis, V. , Landsmanova, J. , Bednarova, B. , Karbanova, J. , Laine, K. , & Rokyta, Z. (2011). Evaluation of the incision angle of mediolateral episiotomy at 60 degrees. International Journal of Gynaecology and Obstetrics, 112(3), 220–224. 10.1016/J.IJGO.2010.09.015 21247571

[nop22160-bib-0028] Kopas, M. L. (2014). A review of evidence‐based practices for management of the second stage of labor. Journal of Midwifery & Women's Health, 59(3), 264–276. 10.1111/JMWH.12199 24850283

[nop22160-bib-0029] Kozhimannil, K. B. , Karaca‐Mandic, P. , Blauer‐Peterson, C. J. , Shah, N. T. , & Snowden, J. M. (2017). Uptake and utilization of practice guidelines in hospitals in the United States: The case of routine episiotomy. Joint Commission Journal on Quality and Patient Safety, 43(1), 41–48. 10.1016/j.jcjq.2016.10.002 28334585

[nop22160-bib-0030] Leclercq, C. , Braund, S. , & Verspyck, E. (2024). Évolution du taux d'épisiotomies et des lésions obstétricales du sphincter de l'anus depuis les recommandations de 2018. Gynécologie Obstétrique Fertilité & Sénologie, 52, 95–101. 10.1016/j.gofs.2024.01.003 38219814

[nop22160-bib-0031] Marcos‐Rodríguez, A. , Leirós‐Rodríguez, R. , & Hernandez‐Lucas, P. (2023). Efficacy of perineal massage during the second stage of labor for the prevention of perineal injury: A systematic review and meta‐analysis. International Journal of Gynaecology and Obstetrics, 162(3), 802–810. 10.1002/IJGO.14723 36808391

[nop22160-bib-0032] Martínez, E. M. L. , Sáez, Z. A. , Sánchez, E. H. , Ávila, M. C. , Conesa, E. M. , & Ferrer, M. B. C. (2021). Perineal protection methods: Knowledge and use. Revista da Escola de Enfermagem da USP, 55, 1–8. 10.1590/1980-220X-REEUSP-2020-0193 34477194

[nop22160-bib-0033] Martínez‐Galiano, J. M. , Arredondo‐López, B. , Hidalgo‐Ruiz, M. , Narvaez‐Traverso, A. , Lopez‐Morón, I. , & Delgado‐Rodriguez, M. (2020). Suture type used for perineal injury repair and sexual function: A randomized controlled trial. Scientific Reports, 10(1), 10553. 10.1038/S41598-020-67659-2 32601329 PMC7324616

[nop22160-bib-0034] Martínez‐Galiano, J. M. , Arredondo‐López, B. , Molina‐Garcia, L. , Cámara‐Jurado, A. M. , Cocera‐Ruiz, E. , & Rodríguez‐Delgado, M. (2019). Continuous versus discontinuous suture in perineal injuries produced during delivery in primiparous women: A randomized controlled trial. BMC Pregnancy and Childbirth, 19(1), 499. 10.1186/S12884-019-2655-2 31842788 PMC6916034

[nop22160-bib-0035] Ministerio de Sanidad y Política Social . (2010). Guía de Práctica Clínica sobre la Atención al Parto Normal . 1–317.

[nop22160-bib-0036] Mohiudin, H. , Ali, S. , Pisal, P. N. , & Villar, R. (2018). Implementation of the RCOG guidelines for prevention of obstetric anal sphincter injuries (OASIS) at two London Hospitals: A time series analysis. European Journal of Obstetrics and Gynaecology and Reproductive Biology, 224, 89–92. 10.1016/j.ejogrb.2018.03.021 29571123

[nop22160-bib-0037] National Institute for Health and Care Excellence . (2014a). Intrapartum care for health apartum care for healthy women and women and babies babies Clinical guideline . Retrieved from https://www.nice.org.uk/guidance/cg190/resources/intrapartum‐care‐for‐healthy‐women‐and‐babies‐pdf‐35109866447557

[nop22160-bib-0038] National Institute for Health and Care Excellence . (2014b). Intrapartum care for healthy women and babies Clinical guideline . 1–96. 31820894

[nop22160-bib-0039] Okeahialam, N. A. , Sultan, A. H. , & Thakar, R. (2023). The prevention of perineal trauma during vaginal birth. *American* . Journal of Obstetrics and Gynaecology, 230(3S), S991–S1004. 10.1016/j.ajog.2022.06.021 37635056

[nop22160-bib-0040] Palau‐Costafreda, R. , García Gumiel, S. , Eles Velasco, A. , Jansana‐Riera, A. , Orus‐Covisa, L. , Hermida González, J. , Algarra Ramos, M. , Canet‐Vélez, O. , Obregón Gutiérrez, N. , & Escuriet, R. (2023). The first alongside midwifery unit in Spain: A retrospective cohort study of maternal and neonatal outcomes. Birth, 50(4), 1057–1067. 10.1111/birt.12749 37589398

[nop22160-bib-0041] Pergialiotis, V. , Bellos, I. , Fanaki, M. , Vrachnis, N. , & Doumouchtsis, S. K. (2020). Risk factors for severe perineal trauma during childbirth: An updated meta‐analysis. European Journal of Obstetrics, Gynaecology, and Reproductive Biology, 247, 94–100. 10.1016/J.EJOGRB.2020.02.025 32087423

[nop22160-bib-0042] Pergialiotis, V. , Vlachos, D. , Protopapas, A. , Pappa, K. , & Vlachos, G. (2014). Risk factors for severe perineal lacerations during childbirth. International Journal of Gynaecology and Obstetrics, 125(1), 6–14. 10.1016/J.IJGO.2013.09.034 24529800

[nop22160-bib-0043] Shinozaki, K. , Suto, M. , Ota, E. , Eto, H. , & Horiuchi, S. (2022). Postpartum urinary incontinence and birth outcomes as a result of the pushing technique: A systematic review and meta‐analysis. International Urogynecology Journal, 33(6), 1435–1449. 10.1007/S00192-021-05058-5 35103823 PMC9206626

[nop22160-bib-0044] Technical Working Group , & World Health Organization . (1997). Care in normal birth: A practical guide. Birth, 24(2), 121–123. 10.1111/J.1523-536X.1997.00121.PP.X 9271979

[nop22160-bib-0045] Thiagamoorthy, G. , Johnson, A. , Thakar, R. , & Sultan, A. H. (2014). National survey of perineal trauma and its subsequent management in the United Kingdom. International Urogynecology Journal, 25(12), 1621–1627. 10.1007/S00192-014-2406-X 24832856

[nop22160-bib-0046] Verghese, T. S. , Champaneria, R. , Kapoor, D. S. , & Latthe, P. M. (2016). Obstetric anal sphincter injuries after episiotomy: Systematic review and meta‐analysis. International Urogynecology Journal, 27(10), 1459–1467. 10.1007/S00192-016-2956-1 26894605 PMC5035659

[nop22160-bib-0047] Woldegeorgis, B. Z. , Obsa, M. S. , Tolu, L. B. , Bogino, E. A. , Boda, T. I. , & Alemu, H. B. (2022). Episiotomy practice and its associated factors in Africa: A systematic review and meta‐analysis. Frontiers in Medicine, 9, 905174. 10.3389/fmed.2022.905174 35865171 PMC9295659

[nop22160-bib-0048] World Health Organization . (2018). Intrapartum care for a positive childbirth experience . 30070803

[nop22160-bib-0049] Zizzi, P. T. , Trevisan, K. F. , Leister, N. , Cruz, C. D. S. , & Riesco, M. L. G. (2017). Women's pelvic floor muscle strength and urinary and anal incontinence after childbirth: A cross‐sectional study. Revista da Escola de Enfermagem da USP, 51, 1–8. 10.1590/S1980-220X2016209903214 28403368

