# Correction to “Midwives’ approach to the prevention and repair of obstetric perineal trauma in Spain”

**DOI:** 10.1002/nop2.2209

**Published:** 2024-06-10

Laderas Díaz E, Rodríguez‐Almagro J, Picón Rodríguez R, Martínez Galiano JM, Martínez Rodríguez S, Hernández‐Martínez A. Midwives' approach to the prevention and repair of obstetric perineal trauma in Spain. *Nurs Open*. 2024;11(4):e2160. doi:10.1002/nop2.2160

In the ‘AUTHOR CONTRIBUTIONS’ section, the author Juan Miguel Martínez Galiano does not appear. The following text should be included as follows: ‘Juan Miguel Martínez Galiano: Conceptualization, data curation, investigation, methodology, resources, validation, visualization and writing—review & editing’.

Similarly, the following text ‘The authors abide by the copyright terms and conditions of Elsevier and the Australian College of Midwives’ would have to be deleted.

We apologize for this error.

